# Measurement properties of the short version of the Western Ontario and McMaster Universities Arthritis Index (WOMAC) for individuals with knee osteoarthritis

**DOI:** 10.1186/s12891-023-06696-0

**Published:** 2023-07-14

**Authors:** José Edson França da Silva Júnior, Almir Vieira Dibai-Filho, Inaê Silva Santos, Jhonata Botelho Protázio, José Djalma Arrais Júnior, Daniella Dias de Oliveira, Patrícia Gabrielle dos Santos, Cid André Fidelis-de-Paula-Gomes

**Affiliations:** 1 Postgraduate Program in Rehabilitation Sciences, Nove de Julho University, Rua Vergueiro, 235/249, Liberdade, São Paulo, SP CEP 01504-001 Brazil; 2 Postgraduate Program in Physical Education, Universidade Federal do Maranhão, São Luís, MA Brazil

**Keywords:** Reproducibility of results, Knee osteoarthritis, Pain measurement, Chronic pain, Surveys, And questionnaires

## Abstract

**Background:**

Assessment instruments play an essential role in the management of knee osteoarthritis. This study aimed to verify the clinimetric properties and validate the short version of WOMAC’s (SV-WOMAC) knee with two domains, pain (four items) and physical function (eight items) in individuals with knee osteoarthritis (KO).

**Methods:**

Reliability and internal consistency Construct, criterion validity, Ceiling, and floor effects analyses were performed. In addition to the SV-WOMAC, the following instruments were used: the numerical rating scale (NRPS), International Knee Documentation Committee (IKDC), the Short Form Health Survey (SF-36), and WOMAC’s original version. Spearman’s correlation coefficient (rho) was used to determine the magnitude of the correlation between the AFAQ and the other instruments. Moreover, the test–retest reliability and internal consistency were assessed using the intraclass correlation coefficient (ICC) and Cronbach’s alpha, respectively. In addition, standard error of measurement (SEM) and minimum detectable change (MDC) were calculated.

**Results:**

One hundred and thirteen subjects with KO were included for validity analysis, and a subsample of 53 subjects was used for test-retest reliability. Adequate reliability and internal consistency were observed with ICC ≥ 0.76, SEM ≤ 1.85, MDC ≥ 5.1, and Cronbach’s alpha ≥ 0.84. Regarding construct validity, correlations greater than 0.50 were observed with the IKDC, NRPS, and functional domains of the SF-36. The SV-WOMAC showed a correlation > 0.70 with the original version and did not show ceiling and floor effects.

**Conclusion:**

The SV-WOMAC knee has adequate measurement properties to analyze pain and physical function in Brazilian individuals with KO.

## Introduction

With risk factors such as obesity, advanced age, female gender, previous traumatic joint injury, joint malalignment, genetic predisposition, and loss of muscle mass, osteoarthritis is a multifactorial disease related to genetic, hormonal, mechanical, metabolic, and aging factors and characterized initially by molecular disorders (abnormal metabolism of joint tissue) followed by anatomical and/or physiological conditions (degradation of articular cartilage in synovial joints, thickening of the joint capsule, subchondral bone sclerosis, formation of marginal osteophytes, joint inflammation, and loss of function of the affected joint). Although it can affect any joint, the hand, hip, and knee joints are the most affected [1–5].

Due to all this complexity and miscellany of causes and impairments, Patient-reported outcome measures (PROMs) and functional tests have become, over the years, essential components in the assessment of osteoarthritis, especially when it comes to knee osteoarthritis (KO). PROMs express and reflect the individual’s experiences that often cannot be observed by direct measurements [6].

Among the PROMs most used in individuals with KO, the following stand out: the International Knee Documentation Committee (IKDC) which assesses improvement or worsening in symptoms, function, and sports activities; the Medical Outcome Study 36 – Item Short-Form Health Survey SF-36 assesses the quality of life; the numeric rating scale (NRPS) that assesses pain; the Knee Injury and Osteoarthritis Outcome Score (KOOS), which evaluates the quality of life-related to the knee, pain, symptoms, difficulties in performing sports activities and activities of daily living; Knee Injury and Osteoarthritis Outcome Score Physical Function Short Form (KOOS-PS) which assesses physical function and the WOMAC (Western Ontario and McMaster Universities Osteoarthritis Index) which assesses pain, stiffness and physical function [7].

Some studies assessed WOMAC’s structural validity and used factorial and Rasch analysis. According to Gandek [8], when carrying out the factorial analysis, five studies observed a variation of 3 to 7 in the number of WOMAC domains, different from the 3 domains indicated in the original version of the instrument. Bilbao et al. [9] observed that the structure of the Spanish version of WOMAC with 3 domains and 24 items needs to be revised and proposed a short version composed of 2 domains and 11 items, verified through confirmatory factor analysis. Rothenfluh et al. [10] proposed a German version of the WOMAC with 1 domain and 12 items, verified with the Rasch analysis. Davis et al. [6] proposed an English version of the WOMAC with 2 domains and 17 items, verified by Rasch analysis.

Regarding Brazil, the study that carried out the translation, cross-cultural adaptation, and validation of the original version of the WOMAC’s knee into Brazilian Portuguese used the original English version of the instrument, with 3 domains as a basis and obtained adequate values for the reliability and construct validity and is currently used for research and clinical evaluations of individuals with knee and hip osteoarthritis in Brazil [11]. However, this study comes from a master’s thesis and has not gone through a peer review process for publication in any scientific journal. Understanding this scenario, the rationale for testing the properties of the Brazilian Portuguese SV- WOMAC knee came from the results of Ferreira et al. [12], who carried out the structural validity. And unlike the original version, with 24 items and 3 domains, it was reported that the best version of the WOMAC Knee with 2 domains and 12 items [12]. After these results, it became necessary to carry out this study so that it would be possible to verify, through adequate and solid clinometric bases, whether this questionnaire provides reliable data on the pain and physical function of individuals with knee osteoarthritis.

This study aimed to verify the clinimetric properties and validate the SV- WOMAC knee with two domains, pain (four items) and physical function (eight items) in individuals with KO. We hypothesized a magnitude of correlation greater than 0.50 (similar construct) of the SV- WOMAC knee with the original version of the WOMAC Knee, NRPS, IKDC, and SF 36, between 0.30 and 0.50 with the non-functional capacity domain of the SF -36 (related but different constructs), and less than 0.30 with the others SF-36 domains (unrelated constructs).

## Methods

### Study design and ethical considerations

This questionnaire validation study was carried out according to the Guidelines for the Process of Cross-cultural Adaptation of Self-Report Measures and Consensus-based Standards for the Selection of Health Measurement Instruments (COSMIN) [13, 14].

Research participants were recruited by verbal invitation and phone from a list of participants in the care group for individuals with chronic musculoskeletal pain in basic healthcare units, clinics, and healthcare offices in the city of São Paulo (SP, Brazil) between January 2020 and ended in October 2022. Data was collected using the free Google Forms platform (Mountain View, CA, USA). The assessment instruments were self-reported. The responsible researcher was present throughout the completion of the evaluation instruments to clarify doubts about the completion.

All participants signed an informed consent form. This study was approved by the Research Ethics Committee of Universidade Nove de Julho (nº 32675720.9.0000.5511).

### Participants

The sample size was established according to the COSMIN recommendation of 7 times the number of items in the questionnaire, provided that this value is not less than 100 participants [14].

Thus, the following inclusion criteria were considered: Individuals with KO diagnosed by physicians specializing in osteoarthritis, both sexes, aged over 40 years, Kellgren-Lawrence radiologic scale grades 2 and 3, experiencing knee pain ≥ 3 on the visual analog scale, complaint of pain and/or change in knee function lasting ≥ 12 weeks, morning stiffness and those with native language, Brazilian Portuguese, literate, able to read and write in Brazilian Portuguese. The exclusion criteria included Mini-Mental State Examination (MMSE) score below cutoff values (highest education: score ≤ 23 and lowest education: score ≤ 17) [15], acute infectious diseases, fever, tumor or cancer patients, pregnant, significant abnormalities or paresthesia, and previous surgery in the knee or hip with the total or partial prosthesis of the knees or hips.

### Assessment of measurement properties

The measure properties of structural validity, construct validity, and test-retest reliability were chosen to analyze the SV- WOMAC knee. For this, two applications of the SV- WOMAC were performed with an interval of 1 week between applications. This interval was long enough to avoid memory bias and short enough to ensure no changes were found in the measured construct [14, 16, 17]. In addition to validating the construct, the following instruments were applied: the numeric rating scale (NRPS), International Knee Documentation Committee (IKDC), Short Form Health Survey (SF 36), and WOMAC.

Regarding the instruments used, the NRPS is validated in Portuguese to assess pain intensity. Composed of a sequence of numbers ranging from 0 (no pain) to 10 (worst pain imaginable) [18]. It was established that the pain intensity evaluated was based on the last 7 days of the evaluation [18].

The IKDC was validated for the Brazilian population [19], an instrument developed to detect improvement or worsening in symptoms, functions, and sports activities related to a knee impairment. For this, it has 3 domains and 18 items, namely: Symptoms with 7 items, Sports and daily activities with 10 items (1 for sports and 9 for daily activities), 2 items for knee function (1 item for post-injury function and 1 item for pre-injury function, which is not valid for the composition of the total score. The answer options vary for each item. Item 6 dichotomizes the answer into yes/no; items 1, 4, 5, 7, 8, and 9 use a 5-point Likert scale, and items 2, 3, and 10 11 points. The scores for each item are added to give a total score (excluding the item on the pre-injury function). The possible score range is from 0 to 100; the closer to 100, the lower the limitation involving daily activities or sports and the lower the presence of symptoms [19, 20].

The SF-36 was translated, cross-culturally adapted to Brazilian Portuguese, and is considered valid and reproducible. It aims to assess the quality of life [21]. This is why it is often used to verify the construct validity of other questionnaires [21]. The SF-36 evaluates 8 domains: functional capacity, physical aspects, pain, general health status, vitality, social aspects, emotional aspects, and mental health. The score of each domain is summed in a score from 0 to 100, in which 0 corresponds to the worst health status and 100 to the best health status [21].

The WOMAC is one of the most used instruments for evaluating individuals with knee osteoarthritis. Translated and cross-culturally adapted into Brazilian Portuguese, it contains 24 items divided into three domains: pain with 5 items, stiffness with 2 items, and physical function with 17 items. For each item, a Likert scale with 5 answers is used (none = 0, little = 1, moderate = 2, intense = 3, and very intense = 4). The higher the score, the greater the pain, stiffness, or worse the individual’s physical function [11, 22].

The SV-WOMAC knee resulted from the factor analysis (exploratory and confirmatory) of the original version of WOMAC [12]. This version has 12 items divided into two domains: pain with 4 items and physical function with 8 items, totaling 48 points. The higher the score, the greater the pain or, the worse the individual’s physical function [12].

### Statistical analysis

Descriptive statistics were performed, and variables were presented as mean and standard deviation (SD) or absolute and relative frequency. SPSS software (version 17.0, Chicago, IL, USA) was used for descriptive statistics, reliability, internal consistency, and construct validity analyses.

Therefore, the internal consistency was calculated using Cronbach’s alpha to identify whether redundant or heterogeneous items were in the questionnaire. We considered adequate value on Cronbach’s alpha > 0.70 [16]. We evaluated the reliability using a test–retest model using the intraclass correlation coefficient (ICC). We considered adequate value on ICC > 0.75 [23]. In addition, we calculated the standard error of measurement (SEM) and minimum detectable change (MDC) [24].

On the construct validity, we used Spearman’s correlation coefficient (rho) to determine the correlation magnitude between the SV- WOMAC knee and the other instruments. Our hypothesis is a correlation greater than 0.50 between WOMAC versions. And NRPS, IKDC, and SF-36 (functional capacity domain), between 0.30 and 0.50 and less than 0.30 with the other SF-36 domains.

Ceiling and floor effects were evaluated. This, by definition, occurs when more than 15% of the study participants reach the minimum or maximum values of the final score of the questionnaire evaluation instrument [25].

## Results

### Sample characteristics

We included one hundred-three participants in the study. Fifty participants were used to calculate the construct and criterion validity and to verify the presence of ceiling and floor effects. The data from the fifty-three were used to calculate the reliability and internal consistency.

Most of the sample comprises females, elderly, married, and with complete secondary education, as shown in Table 1.

**Table 1:** Personal characteristics of the study sample (*n* = 103)

Characteristics	Mean (standard deviation) or n (%)
Age (years)	60.64 (10.04)
Weight (Kg)	78.33(15.4)
Height (m)	1.64(6,23)
BMI (Kg/m^2^)	28,78(6,34)
Sex	
Male	7 (6.80%)
Felame	96 (93.20%)
Marital status	
Single	12 (11.65%)
Married	53 (51.45%)
Divorced	17 (16.50%)
Widower	21 (20.40%)
Evel of education	
Complete primary education	11 (10.67%)
Incomplete secondary education	15 (14.56%)
Complete secondary education	38 (36.90%)
Incomplete higher education	15 (14.56%)
Complete higher education	16 (15.55%
Complete postgraduate	8 (7.76%)
Professional activity	
Active	67 (65.05%)
Inactive	36 (34.95%)
Affected limb	
Right	39
Left	43
Bilateral	21
Diagnostic time (Years)	11,02
NRPS	6,16(2,53)
SV – WOMAC knee	
Pain	7,50(3,55)
Physical Function	15,37(7,48)
WOMAC	
Pain	9,42 (4,54)
Stiffness	4,0 (2,01)
Physical Function	32,17 (13,37)
IKDC	33,69(16,82)
SF 36	
Functional capacity	33,49 (26,32)
Physical aspects	33,98 (15,62)
Pain	27,11 (17,41)
General health status	43,39 (22,31)
Vitality	42,91 (13,69)
Social aspects	53,76 (22,87)
Emotional aspects	56,95 (10,62)
Mental health	51,37 (12,54)

*NRPS* Numeric rating scale, *SV-WOMAC* Short version of the Western Ontario and McMaster Universities Arthritis Index, *WOMAC* Western Ontario and McMaster Universities Arthritis Index, *IKDC* International Knee Documentation Committee, *SF 36* Short Form Health Survey

### Reliability and internal consistency

A subsample (*n* = 53) participated in this analysis for reliability and internal consistency analyses. As shown in Table 2. Identified excellent values for reliability with ICC = 0.85 and SEM = 10.58% for the Pain domain. And for the Physical function domain, ICC = 0.76, SEM = 13.21. In addition, the SV- WOMAC knee has adequate internal consistency (Cronbach’s alpha = 0.849 and 0.996, respectively, for pain and physical function domains) (Table 2).

**Table 2:** Reliability and intermittent consistency of the domains of the SV-WOMAC

Variable	Domains and values Mean (standard deviation)	
SV – WOMAC knee	Pain	Physical Function
Test	7,90 (2,21)	14,73 (3,41)
Retest	7,33 (1,95)	13,35 (4,16)
Intraclass correlation coefficient_2,1_ (95% CI)	0,85	0,76
Standard error of measurement, score	0,81	1,85
Standard error of measurement (%)	10,58	13,21
Minimum detectable difference, score	2,23	5,14
Minimum detectable difference (%)	29,32	36,61
Cronbach’s alpha	0,849	0,996

*SV-WOMAC* Short version of the Western Ontario and McMaster Universities Arthritis Index

### Construct and criterion validity

We hypothesized the magnitude of correlation greater than 0.50 (similar construct) of the SV-WOMAC knee with the WOMAC of the knee, IKDC, and SF 36, between 0.30 and 0.50 with the non-functional capacity domain of the SF -36 (related but different constructs), and less than 0.30 with the other research instruments (unrelated constructs).

Thus, we confirmed our hypothesis, as the SV-WOMAC knee presented a correlation magnitude greater than 0.50 with the IKDC with the SF-36 domains and the NRPS (Table 3). Regarding criterion validity, Table 4 shows that the WOMAC short version’s pain and physical function domains presented adequate cutoff values for the magnitude of correlation (> 0.70) between the pain and physical function domains of the original version of the WOMAC.

**Table 3:** Correlation between the SV-WOMAC knee and the other instruments used in this study

Instruments	SV- WOMAC knee	
	Pain	Physical Function
NRPS	0,764**	0,684**
IKDC	-0,836**	-0,737**
SF 36		
Functional capacity	-0,823**	-0,680**
Physical aspects	-0,706**	-0,560**
Pain	-0,799**	-0,763**
General health status	-0,758**	-0,644**
Vitality	-0,555**	-0,522**
Social aspects	-0,705**	-0,707**
Emotional aspects	-0,375**	-0,237**
Mental health	-0,517**	-0,514**

*NRPS* Numeric rating scale, *SV- WOMAC knee* Short version of the Western Ontario and McMaster Universities Arthritis Index, *IKDC* International Knee Documentation Committee, *SF 36* Short Form Health Survey

^**^Significant correlation (*p* < 0.01), * Significant correlation (*p* < 0.05)

**Table 4:** Correlation between short and long versions of WOMAC knee

Instruments	SV- WOMAC knee Pain	SV- WOMAC knee Physical Function
WOMAC		
Pain	0,938**	0,843**
Stiffness	0,659**	0,656**
Physical Function	0,908**	0,841**

*WOMAC* Western Ontario and McMaster Universities Arthritis Index

### Ceiling and floor effects

No ceiling and floor effects were observed. In the short version of the SV- WOMAC knee, 1 (1%) participant achieved the minimum score, while 2 (1.9%) completed the maximum score. Therefore, in the physical function domain of the SV-WOMAC knee, none of the participants reached the minimum score, while 2 (1.9%) reached the maximum score.

## Discussion

This study identified that the short Brazilian Portuguese version of the SV- WOMAC knee has adequate measurement properties. Precisely, this version has adequate values for reliability, internal consistency, construct validity, and criterion validity, not showing ceiling and floor effects. Characteristics that make this version a reliable assessment tool to assess pain and physical function in individuals with KO.

The hypothesis tested in the study was based on the magnitude of correlation greater than 0.50 between WOMAC versions. And NRPS, IKDC, and SF-36 (functional capacity domain), between 0.30 and 0.50. And less than 0.30 with the other SF-36 domains. Indeed, the tested hypothesis was confirmed. It indicated that the SV-WOMAC has a great magnitude of correction with the original instrument and a great relationship with instruments that evaluate recurrently indicated constructs for assessing KO, pain intensity, and functionality [6, 7]. The lowest correlation magnitude concerned the emotional aspects domain. This result is consistent with the WOMAC composition structure, which does not directly assess emotional aspects.

The results of Ferreira et al. [12] and this research deepen and improve the discussion on the WOMAC Portuguese knee in Brazil. The WOMAC is one of the most used instruments in research and clinical practice regarding the knee joint [6]. However, the Brazilian Portuguese version was based on a study without publishing the results in scientific journals [11]. Years later, Lage et al. [26] carried out a cohort (ELSA-Brasil Musculoskeletal Cohort) that attested to the good quality of WOMAC properties. However, it is noteworthy that individuals with non-specific knee pain were included for this cohort to be carried out. However, the authors emphasize that the variability in measurement properties in different population strata must be considered when using the Womac. For this very reason, aiming at a more homogeneous and precise analysis, the same inclusion criteria used by Ferreira et al. [12] were used to carry out this study, with a sample composed exclusively of individuals diagnosed with KO.

Because it is understood that the establishment of the measurement properties of an instrument within a group of individuals is a continuous and changing process,50 studies questioning the measurement properties related to the WOMAC are not exclusive to the Brazilian Portuguese version [12]. McConnell et al. [27] reported that preliminary evidence supports the use of WOMAC in groups of orthopedic patients, except those with hip and knee osteoarthritis. Much because the subscale to assess joint stiffness has limited evidence to support its use McConnell et al. [27], mainly because the measurement properties of this subscale have yet to be well demonstrated in the literature. The stiffness subscale showed good internal consistency, but the test-retest reliability exceeded expectations. Therefore, its convergent validity, involving individuals with hip and knee osteoarthritis [27], is scarce.

The findings by McConnell et al. [27] reinforce the use of the Brazilian Portuguese SV- WOMAC knee proposed by Ferreira et al. [12]. Mainly because, in this short version, the joint stiffness subscale did not show adequate properties and was excluded from the instrument. However, excluding or not applying the subscale would not solve the problem since it would not be possible to calculate the final global score of the WOMAC [27]. It was essential to present a version with adequate measurement properties to support its application.

Reduced versions of WOMAC are not exclusive to the Brazilian Portuguese version. Short questionnaires improve patient compliance and present more reliable response rates [9]. As a result, a short version of the WOMAC, for the hip, in Spanish was proposed and validated. Even though it is a version aimed at the hip, specifically for hip osteoarthritis submitted to total hip arthroplasty. The final version of the short WOMAC in Spanish had 11 items, and, like the version used in this study, the joint stiffness domain was also excluded.

As for internal consistency, Collins et al. [7] found Cronbach’s α values between 0.67–0.92 for the pain domain and 0.82–0.98 for the physical function domain in studies that validated the original version of the WOMAC’s knee. Values close to those obtained by our version, which reached 0.84 for the pain domain and 0.99 for the physical function domain. A systematic review by Gandek [8] investigated the measurement properties of the WOMAC, analyzing 76 validation and cross-cultural adaptation studies. It was attested that a good internal consistency should be considered when values above 0.70 of Cronbach’s α are reached in the pain domain and above 0.90–0.95 in the physical function domain [8]. With this, it can be attested that the SV-WOMAC knee Portuguese from Brazil obtained excellent internal consistency, with Cronbach’s α values of 0.84 in the pain domain and 0.99 in the physical function domain.

Regarding the construct validity, the Brazilian Portuguese SV- WOMAC knee presented an excellent correlation magnitude with all other instruments used for comparison in this study. As a reference, a valid correlation is above 0.50 compared to other instruments with similar constructs [25]. The cutoff point was reached in correlation with the IKDC, the NRPS, and the functional domains of the SF-36. Specifically, the non-functional SF-36 domains with related constructs that do not directly analyze pain and physical function have a valid correlation between 0.30 and 0.50, the cutoff point reached by the short version of the SV-WOMAC knee.

Regarding criterion validity, the correlation magnitude of the SV- WOMAC knee was analyzed with the original version; an adequate correlation was attested (> 0.70) in the pain and physical function domains, with no correlation above 0.70 only with the stiffness domain. This finding indicates that even with the deletion of 12 items, the measurement ability of the SV-WOMAC knee for pain and physical function remained very close to the original version. With the advantage of being shorter, with a shorter application time. And consequently, better accuracy.

The validation of the SV- WOMAC knee into Brazilian Portuguese presents a new possibility for clinicians and researchers. Its greater simplicity and ease of application will enhance and increase its acceptability and usefulness in research and clinical management routines of individuals with KO. In clinical management and conducting clinical research, participants often need to fill in several extensive assessment instruments, which implies a significant burden and may compromise responses. It is understood that short questionnaires result in greater patient compliance and better response rates [9].

Some limitations of this study must be recognized. First, the assessment instruments were completed in person, and the digital filling was used. However, instruments such as the WOMAC already have validation to be self-administered or used through interviews, for personal use, by telephone, or electronically (via cell phone or computer) [28–30]. Furthermore, the performance of treatments or interventions such as analgesics, corticosteroids (injectable or not), and non-steroidal anti-inflammatory drugs during the period of participation of individuals in the study was not controlled.

The results demonstrated in this study offer several opportunities for future studies. Although the extended version of WOMAC, which gave rise to the version tested in this study, has a good linking process with the International Classification of Functioning, Disability and Health (ICF) [31, 32]. New studies can be carried out to link SV- WOMAC to the ICF Core Set for KO. Therefore, new studies can analyze the responsiveness of the SV- WOMAC knee. And yet, the verification of SV-WOMAC properties in individuals who have hip osteoarthritis or have undergone hip and knee arthroplasties.

## Conclusion

The SV-WOMAC knee has adequate measurement properties to analyze pain and physical function in Brazilian individuals with KO.

## Data Availability

The datasets generated and/or analyzed during the current study are not publicly available due to our limitation of digital data stores for collective access but are available from the corresponding author at reasonable request.
